# Prioritising wheelchair services for children: a pilot discrete choice experiment to understand how child wheelchair users and their parents prioritise different attributes of wheelchair services

**DOI:** 10.1186/s40814-016-0074-y

**Published:** 2016-07-19

**Authors:** Nathan Bray, Seow Tien Yeo, Jane Noyes, Nigel Harris, Rhiannon Tudor Edwards

**Affiliations:** 1 Centre for Health Economics and Medicines Evaluation, Bangor University, Ardudwy, Normal Site, Bangor, Gwynedd LL57 2PZ UK; 2 School of Social Sciences, Bangor University, Neuadd Ogwen, Bangor, Gwynedd LL57 2DG UK; 3 DesignAbility, Bath Institute of Medical Engineering, The Wolfson Centre, Royal United Hospital, Bath, BA1 3NG UK

**Keywords:** Discrete choice experiment, Health economics, Conjoint analysis, Childhood disability, Wheelchair, Assistive technology

## Abstract

**Background:**

Approximately 95 million children worldwide are disabled; 10 % use a wheelchair. In the UK, an estimated 770,000 children are disabled. National Health Service Wheelchair Services are the largest provider of wheelchairs in the UK; however, recent reports have highlighted issues with these services. This study explores the use of discrete choice experiment methods to inform wheelchair service provision for disabled children based on service user preferences. The aim was to explore how disabled children and their parents prioritise different attributes of wheelchair services. The secondary aims were to compare priorities between parents and disabled children and to explore marginal rate of substitution for incremental changes in attributes.

**Methods:**

Discrete choice experiments are a method of attribute-based stated preference valuation used by health economists to understand how individuals prioritise different attributes of healthcare services and treatments. We conducted the first pilot discrete choice experiment to explore how disabled children (aged 11 to 18) and their parents prioritise different attributes of hypothetical wheelchair services. Eleven disabled children (aged 11 to 18) and 30 parents of disabled children completed eight pairwise choice tasks based on five service attributes: wheelchair assessment, cost contribution, training, delivery time and frequency of review. Data were analysed using conditional logistic regression. For each pairwise choice, the participants were asked to choose which service scenario (A or B) they preferred.

**Results:**

Comprehensiveness of wheelchair assessment and wheelchair delivery time significantly (*P* < 0.05) affected service preferences of children (*β*-coefficients = 1.43 [95 % bootstrapped CI = 1.42 to 2.08] and −0.92 [95 % bootstrapped CI = −1.41 to −0.84], respectively) and parents (*β*-coefficients = 1.53 [95 % bootstrapped CI = 1.45 to 2.16] and −1.37 [95 % bootstrapped CI = −1.99 to −1.31], respectively). Parents were willing to contribute more financially to receive preferred services, although this was non-significant.

**Conclusions:**

Both samples placed the greatest importance on holistic wheelchair assessments encompassing more than health. The National Health Service should consider using discrete choice experiment methods to examine wheelchair service preferences of disabled children (aged 11 and over) and their parents on a wider scale; however, care must be taken to ensure that this method is used appropriately.

**Electronic supplementary material:**

The online version of this article (doi:10.1186/s40814-016-0074-y) contains supplementary material, which is available to authorized users.

## Background

It is estimated that 95 million children worldwide have a disability, 13 million of which have a severe disability [1]. Approximately 10 % of disabled people require a wheelchair to maintain mobility [2]. Independent mobility for disabled people and provision of equipment to facilitate this are considered human rights by the United Nations [3]. Without adequate wheelchair provision, many disabled people are caught in a cycle of poverty and deprivation, with reduced access to education, work and social facilities [4]. It is estimated that 20 million disabled people worldwide do not have access to appropriate wheelchair equipment to maintain mobility and independence [4].

In the UK, there are an estimated 770,000 disabled people under the age of 16 [5], approximately 70,000 of which have unmet mobility needs [6]. The provision of a wheelchair at the appropriate time can offer a range of holistic benefits for disabled children and young people, for instance, functional mobility improvement [7], psychosocial development [8], development of communication skills [7, 9, 10], and increased independence [7, 11].

Common physical disabilities which lead to mobility impairment include cerebral palsy, muscular dystrophy, spinal muscular atrophy and spina bifida. Traditional medical definitions describe disability as a deficit in functional ability, sensation or capacity. This assumes that the causes of disability are therefore a direct result of individual abnormal physical, cognitive or sensory functioning, thus defining disability as a disadvantage and human diversity as a scale from normal to abnormal [12]. The social model of disability focusses on how social oppression and discrimination disable those with impairments. Impairment and disability are therefore defined as two separate concepts; impairment is a bodily state characterised by physical or cognitive malfunction, while disability is the disadvantage or restriction faced by people with impairments due to societal, organisational and/or institutional barriers [13]. Wheelchairs offer a means for people with physical disabilities to alleviate some of their mobility issues and therefore reduce their experience of disability.

In the UK, National Health Service (NHS) Wheelchair Services are the largest provider of wheelchairs to disabled children. However, a number of high-profile inquiries have highlighted inadequacies in NHS wheelchair services for children and young people [14–16]. The National Assembly for Wales Health, Wellbeing and Local Government Committee report [17] found that Welsh NHS wheelchair services were supplying inadequate wheelchair interventions to enable children to lead fulfilled lives. Service development recommendations included reduced waiting times; a more holistic approach to wheelchair provision (taking into account social, educational and developmental outcomes); and improvement of inadequate review procedures and information provision.

At present, there is no published evidence as to how wheelchair service users prioritise different attributes of wheelchair services either explicitly or implicitly; thus, the relative importance of these attributes to service users is not currently known. Disabled children and their parents should be engaged in shaping wheelchair services at the local level [18, 19]. In order for this to be achieved, it is important to understand how service users prioritise the different attributes of wheelchair services.

### Aims and objectives

The primary aim of this pilot study was to explore the preferences of disabled children and their parents for different attributes of wheelchair services. The secondary objectives were as follows:

- To compare the preferences of disabled children and their parents for different attributes of wheelchair services
- To calculate hypothetical marginal rate of substitution (MRS) values for different configurations of wheelchair services using cost contribution as the denominator
- To illustrate the use of discrete choice experiment (DCE) methods with disabled children, in relation to wheelchair services

## Methods

### Ethical considerations

The study was granted ethical approval by the North West Wales NHS Research Ethics Committee (reference: 13/WA/0143) and an academic ethics committee at Bangor University. Eligible participants were sent postal information about the study and indicated their consent to participate by returning a completed demographic questionnaire, after which a date and time for administration of the DCE questionnaire was arranged. The participants were offered a small financial incentive (a £10 retail voucher) for taking part in the study.

Before administering the DCE questionnaire the study was explained in full to the participants, and they were informed of the data collection process and the aims of the research. The participants were then asked to complete a second consent/assent form to indicate that they understood and agreed to take part. Children under the age of 16 completed an assent form, and their parents completed a proxy consent form. Data collection was conducted in the home of the participants where possible.

### Sampling and recruitment

A convenience sample was used. The sampling frame was disabled children (aged 18 or under) who use a wheelchair and their parents. The pilot DCE was part of a larger programme of research called the Wheels Project (funded by the National Institute for Social Care and Health Research), which also included assessment of health-related quality of life. This paper presents only the DCE data.

The participants were recruited between June and October 2013 from three recruitment sites: an NHS wheelchair service, a charity-powered wheelchair (PWC) manufacturer/supplier and a children’s wheelchair charity. The DCE questionnaire was completed as part of a separate interview for the Wheels Project. One parent per child was asked to complete the DCE.

### DCE design

DCEs are an established method of conjoint analysis used in health economics to elicit stated preferences for different services or different attributes of services. Individuals are asked to make decisions between hypothetical scenarios with differing attributes. Their preferences, and trade-offs between levels of attributes, are then inferred based on their patterns of choices. DCEs are therefore a form of attribute-based stated preference valuation. A DCE is designed as a number of hypothetical scenarios arranged into paired choice scenarios. These paired choice scenarios have a set number of attributes (e.g. cost) with varying levels (e.g. £50 or £150) chosen by the researcher based on previous research and expertise. Individuals are asked to make trade-offs between the attributes in the DCE tasks by comparing the variation of levels between pairwise choices and then choosing between the two or more competing hypothetical scenarios, thus revealing their relative preference for different service attributes [20]. The development and reporting of this DCE meets the criteria set out in the ISPOR conjoint analysis checklist [21].

The DCE attributes, levels and design for this pilot study were derived from a mixed-method systematic review of the literature [19] and through discussion with young wheelchair users (aged 11 to 18) and healthcare professionals working within wheelchair services. The systematic review [19] incorporated a range of mixed-method evidence relating to wheelchair services and service users experiences of them. Government reports and policy evidence were thematically coded and synthesised to produce seven emergent categories of wheelchair service priority areas: waiting times; joint working and multi-agency approach; effective use and outcomes; funding and procurement; aftercare and information; eligibility criteria and assessment; and service user involvement. The overarching findings from the systematic review were synthesised to develop a conceptual framework for effective wheelchair service development, which was subsequently used to refine the emergent categories into ten wheelchair service attributes (and their levels) for the DCE.

A list of these ten attributes (and their levels) was presented to healthcare professionals in wheelchair provision (*n* = 5), who were asked to consider which five attributes they felt were most relevant to the current practice and which levels were most reflective of the current practice and service targets. As the DCE was intended to be completed by children, we chose to limit the design to five attributes in order to reduce burden on the respondents. Five attributes from the original list of ten were excluded following consensus from the wheelchair professionals: waiting time for assessment; distance of wheelchair service from patients’ home; patient transport provided by wheelchair service; availability of loan equipment; and cost of maintenance. The remaining five key attributes were as follows: (1) comprehensiveness of wheelchair assessment, (2) cost contribution for wheelchair, (3) level of training provided by service, (4) waiting time for delivery of wheelchair and (5) frequency of wheelchair reviews. Of these five attributes, four were assigned two levels (e.g. wait 1–3 months or 6–12 months for delivery) and one was assigned four levels (e.g. pay nothing, £50, £150 or £300). See Table 1 for a full list of attributes, levels and effect codes. The attribute levels represent both the current NHS practice and aspirational practice:

- Holistic wheelchair assessments (considering important factors such as social and educational needs) have been recommended by a number of government reports, as clinical need has historically been solely used to assess assistive technology needs [14, 17].
- The average cost of a standard manual wheelchair supplied by the NHS is £270 [22]. The NHS is free at the point of care; however, service users can choose to purchase wheelchairs privately through the NHS wheelchair voucher scheme, which allows service users to request a voucher towards the cost of a privately funded wheelchair [23]. The attribute levels were set relatively low in order to reflect a non-prohibitive service user contribution.
- NHS services provide wheelchair skills training as part of standard practice, while charitable organisations, such as Whizz-Kidz, provide advanced wheelchair skills training and life skills training outside of the NHS.
- Delivery time for NHS wheelchairs varies across services, with children often waiting up to a year for a wheelchair [17]; the aspirational target is less than 18 weeks from referral to delivery [24].
- NHS services aim for wheelchair reviews and maintenance to be undertaken at least annually [25], although more frequent reviews and maintenance could be beneficial [24].

**Table 1:** Full list of attributes, levels and effect codes for discrete choice experiment questionnaire (child version)

Attribute	Level	Definition (effect coding)
Comprehensiveness of wheelchair assessment	Health needs	Your health needs will be considered in the wheelchair assessment (0)
	Health, school and social life needs	Your health, school and social life needs will be considered in the wheelchair assessment (1)
Cost (£) contribution for wheelchair	No cost	You will not have to contribute any money for your wheelchair (0)
	£50	You will have to contribute £50 for your wheelchair. This would be a one-off payment for each new wheelchair (50)
	£150	You will have to contribute £150 for your wheelchair. This would be a one-off payment for each new wheelchair (150)
	£300	You will have to contribute £300 for your wheelchair. This would be a one-off payment for each new wheelchair (300)
Level of training provided by service	Wheelchair skills training	You will receive wheelchair skills training as part of the service. Wheelchair skills training will include wheelchair driving techniques, road safety and maintaining your wheelchair (0)
	Wheelchair and life skills training	You will receive wheelchair skills training and life skills training as part of the service. Wheelchair skills training will include wheelchair driving techniques, road safety and maintaining your wheelchair. Life skills training will include work placements, learning independence and ambassador groups (1)
Delivery time for delivery wheelchair	Between 1 and 3 months	It will take between 1 and 3 months for your wheelchair to be delivered after the final assessment (0)
	Between 6 and 12 months	It will take between 6 and 12 months for your wheelchair to be delivered after the final assessment (1)
Frequency of wheelchair review	At least every 6 months	Your needs and wheelchair will be reviewed every 6 months. This will include a reassessment of your needs and a review of your wheelchair for any maintenance or repairs it requires (6)
	At least every 12 months	Your needs and wheelchair will be reviewed every 12 months. This will include a reassessment of your needs and a review of your wheelchair for any maintenance or repairs it requires (12)

Following feedback from wheelchair professionals, a preliminary DCE was designed and presented to a small sample (*n* = 10) of young wheelchair users (aged 11 to 18) at a children’s wheelchair charity beneficiary meeting in order to gauge their understanding of the DCE method and the appropriateness of the attributes, levels and questionnaire design.

**Fig. 1:**
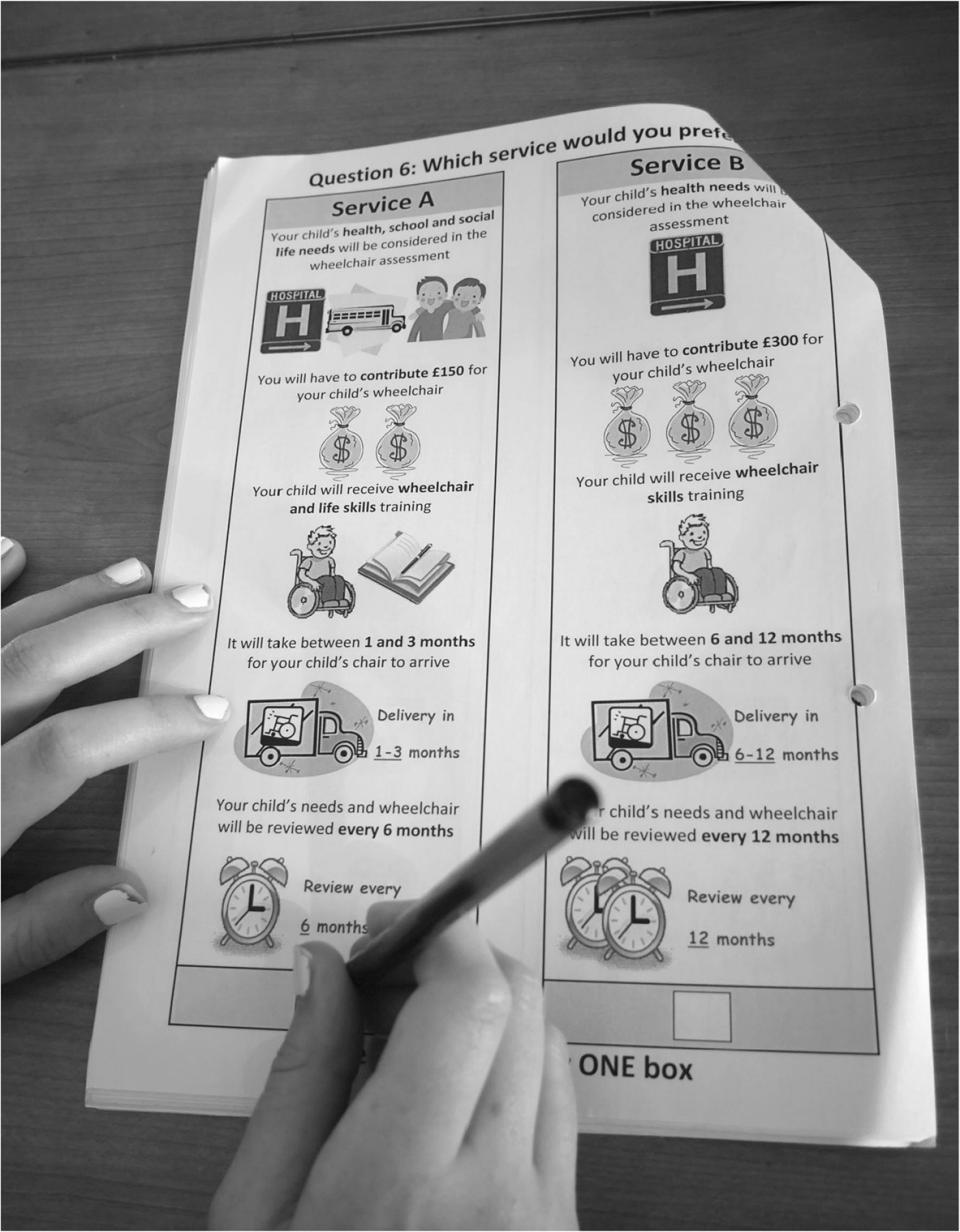
Example of parent DCE questionnaire being completed. Pictorial representations of the DCE attribute and levels, as displayed in the DCE questionnaires completed by children and their parents

Subsequent to the feedback obtained from the beneficiary meeting, the design and layout of the DCE was refined in order to make it easier to understand for children from age 11. This included developing pictorial representations of the attributes and levels to increase ease of use (see Fig. 1). No further changes to the attributes and levels were recommended by the young wheelchair users. Two versions of the DCE questionnaire were developed to allow for slight differences in wording of questions for parents and children, although the content of the tasks remained the same.

The final combination of attributes and levels produced a full factorial design of 64 hypothetical service scenarios. For ease of completion, an appropriate mixed-level orthogonal array was used to reduce the number of scenarios down to eight with efficient design [26]. Coding of attribute levels for the eight scenarios was obtained from an appropriate mixed-level orthogonal array [26]. Scenarios were mirrored and paired in a fold-over design, so that each of the eight scenarios had a mirrored alternative with opposite attribute levels, giving a total of 16 scenarios in eight pairwise choice tasks. This ensured that there was minimum overlap and attribute levels were not repeated across pairwise choices. For each pairwise choice, the participants were asked to choose which of the two hypothetical service scenarios (service A or service B) they preferred. See Table 2 for an example of a pairwise choice task.

**Table 2:** Example of parent DCE pairwise choice task

Service A	Service B
Your child’s health, school and social life needs will be considered in the wheelchair assessment	Your child’s health needs will be considered in the wheelchair assessment
The service will be free	You will have to contribute £50 for your child’s wheelchair
Your child will receive wheelchair and life skills training	Your child will receive wheelchair skills training
It will take between 6 and 12 months for your child’s chair to arrive	It will take between 1 and 3 months for your child’s chair to arrive
Your child’s needs and wheelchair will be reviewed every 6 months	Your child’s needs and wheelchair will be reviewed every 12 months
Which service would you prefer? Please tick one box below:	
	

Instructions on how to complete the DCE questionnaire were presented to the participants at the beginning of the questionnaire. A supplementary notes section was included with the questionnaire for further information on the attributes and levels (see Additional file 1). In most cases, a researcher was present during completion of the DCE to answer any questions. A small number of participants (*n* = 6) were sent the DCE questionnaire via post; therefore, they completed the questionnaire without a researcher present. Subsequent sub-group analysis to examine the effect of researcher presence was not carried out due to the small sub-group size.

### Data analysis

SPSS v20.0 (IBM Corp., Armonk, New York, USA) and Stata v10.1 (StataCorp, College Station, Texas, USA) were used to analyse the data. In the main analyses, the child and parent samples were analysed separately. Data were analysed using logistic regression; the conditional logistic regression model was used [27, 28]. For each given pairwise choice, “1” represents service A chosen, and “0” represents service B chosen. Fixed effects logit modelling was used to analyse these data, with choice of service scenario as the dependent variable. The model represents the utility function of how the analysed sample trade-off between service attributes, by selecting either scenario (A or B) in each given pairwise choice set.

The statistical model presented in Additional file 2 shows the equation that describes the estimated fixed effects conditional logistic regression model. In the model, utility is assumed linear and additive [28]. The model represents a linear in parameters main effects utility function, a classic functional form used widely in previous DCEs [29–31]. The deterministic component of the utility function (Δutility) is a function of the attribute levels between service scenarios, where the coefficients of each attribute and the constant term are estimated in the model. The *β*-coefficients are subsequently summed to estimate utility for each combination of attribute levels. This gives an indication of the relative social value of each potential service scenario, and the potential impact of individual attribute levels.

The model enables an analyst to interpret the output of the regression model to determine three factors; firstly, the importance of attributes, assessed by coefficient significance levels (i.e. *P* value less than 0.05); secondly, the effect of an attribute on utility, assessed by examining the *β*-coefficient of each attribute in which the *β*-coefficient shows change in utility when moving from one attribute’s level to another attribute’s level; and finally, the MRS, used to examine the trade-offs between attributes. Clustering of responses was not included in the model due to the small sample size. Clustering would need to be considered in a full DCE in this population.

Using this model, the magnitude of the *β*-coefficient is relative to the change in utility as a result of change in the attribute’s level. A positive *β*-coefficient indicates that as the level increases, so does the likelihood of a participant choosing it. Likewise, a negative *β*-coefficient indicates that as the level increases, the likelihood of participants choosing it decreases.

We hypothesised that a positive *β*-coefficient would be observed for the comprehensiveness of wheelchair assessment attribute and level of training provided by service attribute, as the participants were expected to prefer to have additional in-depth assessment of needs and additional training. For the other attributes, it was hypothesised that a negative *β*-coefficient would be observed, as the participants were expected to prefer lower cost contribution, shorter waiting time for delivery and more regular wheelchair reviews.

Effects codes were assigned to the attribute levels of all quantitative (continuous) and qualitative (non-continuous) variables in the DCE design. All of the qualitative variables were dichotomous; thus, the effect codes of 0 and 1 were assigned to their levels as appropriate. The difference between the effect codes of a qualitative attribute (when moving from “alternative A” to “alternative B” in each pairwise choice) was therefore calculated as either −1 or +1 and assigned to the corresponding qualitative attribute in the Stata dataset.

Attributes were not directly comparable on the same scale due to a mix of quantitative and qualitative attributes; thus, MRS was calculated to attain common scale for all attributes. This allowed comparison between attributes to be made. MRS is the amount of a given attribute that a person is willing to forgo in order to obtain one additional unit of another attribute. For instance, an individual may be willing to contribute towards the cost of the wheelchair in order to reduce the delay in wheelchair delivery. The cost contribution attribute (quantitatively scaled) was used as the denominator to calculate the MRS for a one-unit change in each of the remaining attributes. By dividing the other attribute coefficients by the cost contribution coefficient, the MRS was indirectly estimated. The 95 % confidence intervals for the *β*-coefficients were estimated using non-parametric bootstrapping methods, ran on 5000 iterations using Stata v10.1. All presented confidence intervals are therefore bootstrapped.

### Implementation

#### Sample and response rates

A total of 125 study invitation packs were distributed across England and Wales by the three recruitment sites. These contained initial questionnaires for parents and disabled children. Parents and disabled children were recruited from the same household where possible and appropriate. Thirty-five initial questionnaires were returned by the parents (28 % response rate), who were then invited to complete the DCE questionnaire. Of that number, 30 parent DCE questionnaires were completed in full (85.7 % response rate).

Of the disabled children who returned an initial questionnaire (*n* = 15), 13 met eligibility criteria (aged >10) and were invited to complete the DCE questionnaire. A total of 11 disabled children completed the DCE questionnaire (84.6 % response rate).

All returned DCE questionnaires were completed in full with no major data omissions.

#### Demographic characteristics

Demographic details of the samples are presented in Tables 3 and 4. In the disabled child sample, 63.6 % (*n* = 7) were male, 63.6 % (*n* = 7) were aged 16 to 18 and 81.8 % (*n* = 9) had cerebral palsy. In the parent sample, 86.7 % (*n* = 26) of the respondents were women, 86.7 % (*n* = 26) were aged between 30 and 49 and 66.7 % (*n* = 20) had a child with cerebral palsy. There is a lack of ethnic diversity in both samples with the vast majority of respondents being white British.

**Table 3:** Demographic characteristics of the disabled child sample (*n* = 11)

Demographic characteristics	Number (%)
Study site	
NHS wheelchair service	2 (18.2)
Children’s wheelchair charity	9 (81.8)
Gender	
Female	4 (36.4)
Male	7 (63.6)
Age	
11–15 years	4 (36.4)
16–18 years	7 (63.6)
Ethnicity	
White British	11 (100)
Education	
High school	4 (36.4)
College	5 (45.5)
University	1 (9.1)
Home schooled	1 (9.1)
Child’s condition	
Cerebral palsy	9 (81.8)
Muscular dystrophy	1 (9.1)
Hemiplegia/stroke	1 (9.1)
Frequency of equipment use	
Most of the time	1 (9.1)
All of the time	10 (90.9)
Type of equipment used	
Manual	3 (27.3)
Manual and powered	8 (72.8)

**Table 4:** Demographic characteristics of the parent sample (*n* = 30)

Demographic characteristics	Number (%)
Study site	
NHS wheelchair service	5 (16.7)
Charity PWC supplier	10 (33.3)
Children’s wheelchair charity	15 (50.0)
Gender	
Female	26 (86.7)
Male	4 (13.3)
Age	
21–29 years	2 (6.7)
30–39 years	14 (46.7)
40–49 years	12 (40.0)
50–59 years	2 (6.7)
Ethnicity	
White British	29 (96.7)
White and Asian	1 (3.3)
Marital status	
Married	23 (76.7)
Co-habiting	3 (10.0)
Single	2 (6.7)
Separated	1 (3.3)
Divorced	1 (3.3)
Annual household Income	
£5000–15,000	3 (10.0)
£16,000–£25,000	5 (16.7)
£26,000–£35,000	3 (10.0)
£36,000–£50,000	10 (33.3)
£51,000 or more	8 (26.6)
Missing	1 (3.3)
Employment status	
Full-time	5 (16.7)
Part-time	12 (40.0)
Unemployed/stay-at-home parent	13 (43.3)
Child’s condition/disability	
Cerebral palsy	20 (66.7)
Spinal muscular atrophy	2 (6.7)
Muscular dystrophy	3 (10.0)
Others	5 (16.5)
Child’s age	
5 years or under	15 (50.0)
6–15 years	10 (33.3)
16–18 years	5 (16.7)
Frequency of child’s equipment use	
A little of time	1 (3.3)
Some of the time	6 (20.0)
Most of the time	4 (13.3)
All of the time	18 (60.0)
Missing	1 (3.3)
Type of equipment used by child	
Powered	2 (6.7)
Manual	10 (33.3)
Manual and powered	17 (56.7)
Waiting for first wheelchair	1 (3.3)

## Results

### DCE results: disabled child sample

Table 5 shows the results for the two samples. The disabled child sample results show that the *β*-coefficients of three of the five attributes were statistically significant (*P* < 0.05): comprehensiveness of wheelchair assessment (*β*-coefficient = 1.4247 [*P* = 0.009; 95 % bootstrapped CI = 1.42 to 2.08]), waiting time for delivery of wheelchair (*β*-coefficient = −0.9221 [*P* = 0.041; 95 % bootstrapped CI = −1.41 to −0.84]) and cost contribution for wheelchair (*β*-coefficient = −0.0093 [*P* = 0.019; 95 % bootstrapped CI = −0.014 to −0.009]). The remaining two attributes were non-significant: level of training provided by service (*β*-coefficient = 0.0306 [*P* = 0.924; 95 % bootstrapped CI = −0.20 to 0.29]) and frequency of wheelchair reviews (*β*-coefficient = 0.0364 [*P* = 0.519; 95 % bootstrapped CI = 0.00 to 0.08]). Of the attributes that were significant, comprehensiveness of wheelchair assessment was of greatest importance (*β*-coefficient = 1.4247), followed by waiting time for delivery of wheelchair (*β*-coefficient = −0.9221) and cost contribution for wheelchair (*β*-coefficient = −0.0093). Preference was shown for comprehensive wheelchair assessments (of health, education and social needs), shorter waiting time for delivery of wheelchair and lower cost contribution. For the remaining two non-significant attributes, disabled children preferred (if everything being equal) wheelchair and life skills training and less frequent wheelchair reviews.

**Table 5:** Results from conditional logistic regression model: disabled child study sample (*n* = 11) and parent study sample (*n* = 30)

Attribute	Disabled child sample (*n* = 11)					Parent sample (*n* = 30)				
	*β*-coefficient	95 % bootstrapped CI^a^	*P* value	MRS values^b^ (cost)	95 % bootstrapped CI^a^	*β*-coefficient	95 % bootstrapped CI^a^	*P* value	MRS values^b,c^ (cost)	95 % bootstrapped CI^a^
Comprehensiveness of wheelchair assessment	1.4247*	1.4153 to 2.0824	0.009	£153.19	£133.20 to £182.53	1.5329*	1.4507 to 2.1633	<0.0001	£547.46	£353.38 to £1435.45
Cost contribution for wheelchair	−0.0093*	−0.0138 to −0.0089	0.019	–	–	−0.0028	−0.0060 to 0.0005	0.092	–	–
Level of training provided by service	0.0306	−0.1955 to 0.2858	0.924	–	–	−0.1557	−0.4002 to 0.0311	0.371	–	–
Waiting time for delivery of wheelchair	−0.9221*	−1.4086 to −0.8442	0.041	£99.15	£81.93 to £121.32	−1.3699*	−1.9859 to −1.3104	0.000	£489.25	£313.29 to £1326.78
Frequency of wheelchair reviews	0.0364	−0.0022 to 0.0749	0.519	–	–	−0.0390	−0.0813 to 0.0032	<0.0001	–	–
Number of observations = 88						Number of observations = 240				
Number of individuals = 11						Number of individuals = 30				
Log likelihood function = −26.64						Log likelihood function = −64.51				
Log likelihood ratio (5) = 33.85						Log likelihood ratio (5) = 114.86				

*Significant attribute [*P* < 0.05]

^a^95 % confidence intervals generated using non-parametric bootstrapping (5000 replications)

^b^Marginal rate of substitution values = *β*-coefficient for attribute/*β*-coefficient for cost attribute

^c^Though the cost contribution attribute was not significant to parents (*P* = 0.092 [>0.05]), everything being equal, parents preferred lower cost contribution; the parents’ MRS values were calculated using the cost contribution attribute as the denominator to show how parents trade-off the cost contribution attribute against the other attributes. This allowed comparison with the disabled child sample MRS values

### DCE results: parent sample

The parent sample results show that the *β*-coefficients of two of the five attributes were statistically significant (*P* < 0.05). These were comprehensiveness of wheelchair assessments (*β*-coefficient = 1.5329 [*P* < 0.0001; 95 % bootstrapped CI = 1.45 to 2.16]) and waiting time for wheelchair delivery (*β*-coefficient = −1.3699 [*P* < 0.0001; 95 % bootstrapped CI = −1.99 to −1.31]). This indicates that these two attributes were significant factors in parental choices. The remaining three attributes were non-significant: cost contribution for wheelchair (*β*-coefficient = −0.0028 [*P* = 0.092; 95 % bootstrapped CI = −0.01 to 0.00]), level of training provided by service (*β*-coefficient = −0.1557 [*P* = 0.371; 95 % bootstrapped CI = −0.40 to 0.03]) and frequency of wheelchair reviews (*β*-coefficient = −0.0390 [*P =* 0.260; 95 % bootstrapped CI = −0.08 to 0.00]). Based on the *β*-coefficients of the significant attributes, comprehensiveness of wheelchair assessment was of greatest importance (*β*-coefficient = 1.5329), followed by waiting time for delivery of wheelchair (*β*-coefficient = −1.3699). Preference was shown for comprehensive wheelchair assessments (of health, education and social needs) and shorter waiting time for delivery of wheelchair. For the remaining three non-significant attributes, parents preferred (if everything being equal) lower cost contribution, basic wheelchair skills training and more frequent wheelchair reviews. As cost contribution was not significant, parental MRS values are not reliable.

### Comparison of disabled child and parent DCE and MRS results

Both samples showed preference for comprehensive wheelchair assessments and shorter wheelchair delivery times, in that order. The cost contribution attribute was only significant for the child sample, who showed preference for lower cost contribution. Although cost contribution was not significant for parents, for comparison purposes, we calculated the parental MRS values. Results show that the MRS values were higher for parents (£547.46 [95 % bootstrapped CI = £353.38 to £1435.45] for wheelchair assessment and £489.25 [95 % bootstrapped CI = £313.29 to £1326.77] for delivery waiting time) than for disabled children (£153.19 [95 % bootstrapped CI = £133.20 to £182.53] for wheelchair assessment and £99.15 [95 % bootstrapped CI = £81.93 to £121.32] for delivery waiting time), suggesting the parent sample placed higher importance on these attributes than the disabled child sample. However, as the cost contribution attribute was not significant for parents, it is difficult to make comparisons with the disabled child data.

The disabled child and parent samples differed in direction of coefficient preference for level of training provided by service and frequency of wheelchair reviews: the *β*-coefficients for both attributes indicate that, everything being equal, parents preferred basic wheelchair skills training (*β*-coefficient = −0.1557) and more frequent wheelchair reviews (*β*-coefficient = −0.0390), while disabled children preferred wheelchair and life skills training (*β*-coefficient = 0.0306) and less frequent wheelchair reviews (*β*-coefficient = 0.0364). However, the *β*-coefficients for these attributes were not significant.

### Sub-group analysis: matched pairs of disabled children and their parents

In order to compare preferences of disabled children and their respective parents, a sub-group analysis was performed using only the data from dyads of disabled children (*n* = 9) and their respective parents (*n* = 9), see Additional file 3. Analyses were conducted separately, and preferences were then compared between related disabled children and parents. A smaller distribution of child age was observed, with all children aged 11 or over (63.6 % [*n* = 7] aged 16 or over).

Similarly to the main analysis, both the disabled child and parent samples showed significant preference for comprehensive wheelchair assessments: *β*-coefficients equalled 1.6194 (*P =* 0.015; 95 % bootstrapped CI = 1.58 to 2.29) and 2.1893 (*P* = 0.010; 95 % bootstrapped CI = 2.21 to 3.26) respectively. The cost contribution attribute was again not significant for the parent sample but was borderline significant for the child sample, who showed preference for lower cost contribution (*β*-coefficient = −0.0095 [*P =* 0.050; 95 % bootstrapped CI = −0.013 to −0.009]). All other attributes were non-significant.

## Discussion

This is the first study to systematically elicit and compare the preferences of disabled children and their parents for different attributes of wheelchair services. The findings illustrate the use of the DCE method in a potentially vulnerable sample of the population for the assessment of healthcare services. If appropriately powered, DCE results can be used as a valuable asset in priority setting, service development and healthcare decision-making, as they allow different attributes of services to be ranked by importance, and their relative monetary value to families calculated using MRS.

For the sample of 11 disabled children and 30 parents of disabled children, comprehensiveness of wheelchair assessment was the most important attribute of wheelchair services, followed by wheelchair delivery time. The results from the disabled child sample indicated that cost contribution was also influential. The remaining two attributes (level of training provided by service and frequency of wheelchair reviews) were not statistically significant (*P* > 0.05) for both samples, and thus, they did not impact service preferences.

Both samples showed preference for services that offered assessments which focused on the health, education and social needs of children, as opposed to just health needs. NHS wheelchair services tend to focus on clinical health needs in wheelchair assessment and provision, which may neglect to consider other important aspects of disabled children’s lives [17].

The MRS values of the wheelchair assessment and delivery time attributes were different for the two samples, with parental MRS values higher for both attributes. This would suggest that the sampled parents were willing to contribute more money to attain preferred service attributes. However, it is important to reiterate that cost contribution was not a significant attribute for the sampled parents, while it was for the sampled disabled children. This may reflect that the parent sample were willing to pay more to obtain the best suited services for their child, thus contribution cost did not influence their preferences. Assumptions regarding MRS should be viewed with caution as adults and children are likely to value money differently.

Identical cost contribution levels were used for both samples, which did not take into account differences in how the two samples value money, particularly as the sampled disabled children would have expected to spend family money rather than their own. In hindsight, sampled parents may have felt that contributing up to £300 for a wheelchair was relatively good value for money compared to purchasing a wheelchair privately, while sampled disabled children may have considered this to be a significant amount of money. It is of note that 60 % (*n* = 18) of sampled parents had a household income of over £36,000 per year, which may have impacted their willingness to contribute financially to receive a preferred services for their child.

A future DCE in this field should consider using considerably higher cost contribution attribute levels to test these issues, for instance, setting the levels at retail prices for different types of wheelchairs (e.g. £500, £1500, £3000), or conversely using a more child-friendly approach to cost contribution, such as proportion of income/pocket money. A larger sample would be needed to enable additional sub-group analyses, such as analysing the effect of household income on preferences and MRS values.

Most of the attribute *β*-coefficient directions are reflective of *a priori* hypotheses, although the coefficient directions for frequency of wheelchair reviews for the disabled child sample and level of training for the parent sample were contradictory to these hypotheses. Although not statistically significant, the coefficient direction for the level of training attribute may indicate that sampled parents did not feel it was the responsibility of wheelchair services to provide life skills alongside wheelchair skills training, or potentially that the provision of life skills training may impact on essential wheelchair skills training. This may be a result of the parent sample being skewed towards parents of younger children, as life skills training is likely to be of most importance to older children who want to develop independence. Future research may benefit from defining life skills training based on age (e.g. play skills for children under 5).

For the frequency of wheelchair reviews attribute, the disabled child sample preferred less frequent reviews, although this was also non-significant. This may indicate that the sampled disabled children did not necessarily see the benefit of more frequent reviews of their needs or they may not enjoy reviews and thus would prefer them to be less frequent.

Comparing the results of the full and sub-sample analyses shows variation in parental preferences for frequency of wheelchair reviews and the significance of delivery time on service preference. The parent sub-sample preferred less frequent reviews (as did their children), while the full sample of parents had preference for more frequent reviews. As half of the parents in the full sample had a child aged 5 or under (*n* = 15), this is not entirely surprising, as younger children need more frequent reviews due to their rapidly changing needs associated with growth and development. Interestingly, only comprehensiveness of assessment was found to be a significant attribute in all samples and thus is the most influential attribute on the service preferences of the participants in this study.

These results are congruent with the findings from previous research and recommendations from government and charitable organisation reports. Wheelchairs are important interventions for disabled children to enhance independence, social inclusion and participation [11, 32–35]. It is thus important that wheelchair provision supports optimised physical, cognitive and social development [36] and that wheelchairs are usable in all places required [25, 36, 37]. A holistic approach to assessment and performance measures should be employed to cater for the clinical, social, educational and lifestyle needs of service users [38, 39], which is reflected in published healthcare standards [33]. In order for disabled children to achieve the best outcomes, wheelchairs must be delivered quickly and within set timelines [15–18, 25, 36, 38, 39]. Children’s needs and their wheelchairs should be reviewed at least annually [25].

From July 2015, central information about the NHS wheelchair service volume, expenditure, access and patient experience will be collected from each wheelchair service by NHS England. This will be used to build a national dataset to improve transparency and consistency across NHS wheelchair services, while meeting local supply and demand needs. Furthermore, a national wheelchair tariff designed to improve uniformity and value for money is currently being piloted before a planned national roll-out by 2017. The results from this pilot DCE study are too limited to directly inform service change in the NHS; however, the results do provide impetus for developing services that are based specifically around service user needs and preferences. This study demonstrates that DCE methods could be utilised on a larger scale to inform both national and local changes to services and potentially to develop the NHS tariff around the service preferences of children and their parents in a systematic manner. For instance, the tariff could be designed to promote provision of wheelchairs which meet clinical, social and educational needs of children.

As an example, a 2010 government report highlighted multiple issues with NHS wheelchair services in Wales, UK [17]. This report was in response to criticism from service users and charitable organisations regarding the failures of wheelchair services for children and adults in Wales, particularly waiting times and strict eligibility criteria. In 2012, the National Assembly for Wales outlined a plan for service change and provided an additional annual budget of £2.2 million to wheelchair services across Wales [40]. It is plausible that a DCE could be used to assess how Welsh wheelchair service users prioritise different attributes of these services and then assign additional funding according to service user preferences. This would enable the opinions of service users to drive investment decisions in wheelchair services. This could also be applied to current budget expenditure through service restructuring and reinvestment decisions.

However, it is difficult to see how policy decisions about service attributes could be made based solely on this type of DCE involving disabled children and their parents (even with adequate numbers and statistical power). If service commissioners were to decide to follow lean principles and strip away attributes of wheelchair services based on the results of a large scale DCE, they would be doing so based on the testimonies of families who potentially had not been exposed to aspects of the service they were being asked to state preference for. DCE data in this context would need to be supplemented with evidence of effect to see if additional service attributes improve age-related outcomes. At present, this data is limited, and thus additional research into many aspects of wheelchair provision for disabled children is needed.

Other methods of preference elicitation and service user engagement should also be considered. For instance, qualitative interviews or focus groups would likely be a simpler method of understanding the views of service users and would yield results with more validity in this context. However, qualitative methods are time consuming when gathering large quantities of data and more difficult to synthesise across the respondents. A well-designed DCE can be completed by all capable service users, and thus broader understanding of preferences across all service users may be achievable. DCEs also offer more insight into how service users trade off different attributes of services, thus revealing the relative importance of attributes. Some service users may be unable to adequately verbalise their preferences, thus forced choice DCEs can be used to reveal preferences that individuals would otherwise be unable to communicate.

In practice, a mixture of methods should be used and triangulated to gain insight, as there are pros and cons to both quantitative and qualitative data. Congruence across data sources would indicate a higher degree of validity and robustness.

### Study limitations

This DCE study is a pilot study with small sample sizes and was designed to illustrate the use of the DCE methodology in this particular population group. It is important to note that all participants completed the DCE questionnaire in full without error or missing data and appeared to understand the instructions given.

Due to the size of the samples and their demographic characteristics, the results are not generalisable to the wider population of disabled children who use wheelchairs and their parents. The samples were relatively self-selective; thus, the important views of disengaged or unmotivated individuals may have been missed. Furthermore, it was not possible to take into account important factors such as condition prognosis, age, cognitive ability, growth and the purpose of mobility equipment (e.g. play versus mobility). In a larger sample, it would be important to examine the impact of these factors on preferences, for instance, the different follow-up requirements of children with relatively static conditions, such as cerebral palsy, compared to children with potentially rapidly progressive conditions, such as muscular dystrophy, or children with physical and cognitive disabilities. All child participants had the cognitive ability to complete the questionnaire; therefore, the child sample findings are not generalisable to children with cognitive impairments. The parent sample was more diverse, with most participants (*n* = 19) representing a child who was unable to complete their own DCE questionnaire due to age and/or cognitive ability. Although the sample size is too small to make wider assumptions about the representativeness of the findings, the application of the method illustrates that when disabled children have capacity, DCE methods can be used appropriately as a means of preference elicitation, and that in circumstances where the child does not have capacity their parent(s) may act as a suitable proxy.

Given the small sample sizes, there is a danger of child age causing aggregation to the mean by, for example, including a parent of a 2-year-old in the same analysis as a parent of an 18-year-old, although the sub-group analysis dealt with this to some extent. The age range of the child sample (11–18 years) could also be considered too vast to draw together the results and make wider conclusions, particularly with such a small sample.

The differences between the child and parent samples in terms of child age-related needs and cognitive development are also difficult to compare, and different outcomes should be expected. Making comparisons between the child and parent samples raises some interesting issues. As a general rule, children are not expected to take full responsibility for what happens in their lives; it is up to their parents to take this responsibility, particularly for young children. It is therefore not surprising that there were differences between child and parent preferences. Conversely, in the sub-group analysis, the child and parent preferences were relatively similar and comparable, particularly in terms of *β*-coefficient directions. This raises some interesting questions as to whether children and parents influenced each other’s preferences or whether they genuinely had a shared sense of service preference.

## Conclusions

The results from this study cannot be generalised to the wider population of disabled children and parents due to the small sample sizes and unrepresentative demographic characteristics. The results indicate that for this cohort of disabled children and their parents, the most important wheelchair service attributes were comprehensiveness of wheelchair assessment and wheelchair delivery time. These results support the findings of previous wheelchair services reports and inquiries. Future research should utilise larger and more representative samples. More research is needed into the effective measurement of outcomes from wheelchair provision, particularly addressing social, education and independence needs of disabled children.

This study illustrates the use of DCE methods to examine wheelchair service preferences of disabled children (aged 11 and over) and their parents. Care must be taken to ensure that the methods are used appropriately, for instance, taking into account the layout, language and presentation of the DCE questionnaire. Consideration of methodological implications is required when comparing child and parent preferences.

## Abbreviations

DCE, discrete choice experiment; MRS, marginal rate of substitution; NHS, National Health Service; PWC, powered wheelchair; UK, United Kingdom

## Additional files

Additional file 1: DCE supplementary notes. Full list of supplementary notes provided to participants to aid completion of the DCE questionnaire. (DOCX 15.0kb)
Additional file 2: DCE fixed effects logit model. Full disclosure of DCE fixed effects logit model used to estimate preferences. (DOCX 13.4kb)
Additional file 3: Sub-group analysis. Results from conditional logistic regression model of disabled child (*n* = 9) and parent (*n* = 9) matched pairs. (DOCX 15.2kb)
