# Supplementary material for: Prioritising wheelchair services for children: a pilot discrete choice experiment to understand how child wheelchair users and their parents prioritise different attributes of wheelchair services
Source: Pilot Feasibility Stud. 2016 Jul 19;2:32. doi: 10.1186/s40814-016-0074-y (PMC5154007; doi:10.1186/s40814-016-0074-y)
Supplement: Additional file 1: — DCE supplementary notes. Full list of supplementary notes provided to participants to aid completion of the DCE questionnaire. (DOCX 15.0kb) [file 40814_2016_74_MOESM1_ESM.docx]

### Additional file 1: DCE supplementary notes

Each service is described in terms of the following aspects:

- **How your wheelchair needs will be assessed**

This describes how you will be assessed for a new wheelchair, particularly which of your needs will be assessed. Your needs are defined as health, social life and school needs in relation to using a wheelchair.

- **Cost (£) of the wheelchair to you and/or your family**

This describes how much you will be asked to contribute to the wheelchair service. This would be a one-off payment for each new wheelchair. If you are still at school your parent or guardian would be asked to pay this.

- **The level of training you will receive**

This describes what sort of training you would be given by the wheelchair service. Wheelchair skills training will include wheelchair driving techniques, road safety and maintaining your wheelchair. Life skills training will include work placements, learning independence and ambassador groups.

- **Length of time it takes to receive your wheelchair**

This describes the length of time it takes for your wheelchair to be delivered after your final assessment.

- **How often your wheelchair and needs will be reviewed**

This describes how often you will receive a full review from the wheelchair service. This will include a reassessment of your needs and a review of your wheelchair for any maintenance or repairs it requires.
