# Supplementary material for: Prioritising wheelchair services for children: a pilot discrete choice experiment to understand how child wheelchair users and their parents prioritise different attributes of wheelchair services
Source: Pilot Feasibility Stud. 2016 Jul 19;2:32. doi: 10.1186/s40814-016-0074-y (PMC5154007; doi:10.1186/s40814-016-0074-y)
Supplement: Additional file 2: — DCE fixed effects logit model. Full disclosure of DCE fixed effects logit model used to estimate preferences. (DOCX 13.4kb) [file 40814_2016_74_MOESM2_ESM.docx]

### Additional file 2: DCE fixed effects logit model used to estimate preferences

Δ Utility = α + β1Assess + β2CostCon + β3LvlTrain + β4DelTime + β5FreqRev+ ε1 + ε2

Model definitions:

| Δ Utility  α  β1 - β5  Assess  CostCon  LvlTrain  DelTime  FreqRev  ε1  ε2 | =  =  =  =  =  =  =  =  = | The change in utility in moving from scenario A to scenario B  Constant term  The beta coefficients of the model to be estimated  The difference in comprehensiveness of assessment between scenario A and scenario B  The difference in cost contribution between scenario A and scenario B  The difference in level of training between scenario A and scenario B  The difference in delivery waiting time between scenario A and scenario B  The difference in frequency of review between scenario A and scenario B  The error term because of differences amongst observations  The error term because of differences amongst respondents |
| --- | --- | --- |
