# Supplementary material for: Prioritising wheelchair services for children: a pilot discrete choice experiment to understand how child wheelchair users and their parents prioritise different attributes of wheelchair services
Source: Pilot Feasibility Stud. 2016 Jul 19;2:32. doi: 10.1186/s40814-016-0074-y (PMC5154007; doi:10.1186/s40814-016-0074-y)
Supplement: Additional file 3: — Sub-group analysis. Results from conditional logistic regression model of disabled child (n = 9) and parent (n = 9) matched pairs. (DOCX 15.2kb) [file 40814_2016_74_MOESM3_ESM.docx]

|  | **Disabled child sample (n=9)** | | | | | | |  | **Parent sample (n=9)** | | | | | | |
| --- | --- | --- | --- | --- | --- | --- | --- | --- | --- | --- | --- | --- | --- | --- | --- |
| **Attribute** | **β-coefficient** | | **95% CI**** | ***P*-value** | **MRS values*** (cost)** | **95% CI**** | |  | **β-coefficient** | **95% CI**** | | ***P*-value** | | **MRS values*****^△^ **(cost)** | **95% CI**** |
| Comprehensiveness of wheelchair assessment | 1.6194* | | 1.5745 to 2.2935 | 0.015 | £170.46 | £150.41 to £201.86 | |  | 2.1893* | 2.2088 to 3.2599 | | 0.010 | | £308.35 | £252.53 to £472.02 |
| Cost contribution for wheelchair | -0.0095^▲^ | | -0.0139 to -0.0087 | 0.050 | -- | -- | |  | -0.0071 | -0.0192 to 0.0049 | | 0.246 | | -- | -- |
| Level of training provided by service | -0.1998 | | -0.4645 to 0.0210 | 0.588 | -- | -- | |  | -0.3498 | -1.2170 to 0.5176 | | 0.429 | | -- | -- |
| Waiting time for delivery of wheelchair | -1.0104 | | -2.0454 to 0.0246 | 0.056 | -- | -- | |  | -1.2671 | -2.5926 to 0.0713 | | 0.064 | | -- | -- |
| Frequency of wheelchair reviews | 0.0433 | | -0.0813 to 0.1680 | 0.495 | -- | -- | |  | 0.0040 | -0.0452 to 0.0429 | | 0.955 | | -- | -- |
|  |  | |  |  |  |  | |  |  | |  |  | |  |  |
| Number of observations= 72 | | |  |  |  |  | | | Number of observations= 72 | | | |  |  |  |
| Number of individuals = 9 | | |  |  |  |  | | | Number of individuals = 9 | | | |  |  |  |
| Log likelihood function = -22.51 | | |  |  |  |  | | | Log likelihood function = -16.84 | | | |  |  |  |
| Log likelihood ratio (5) = 27.38 | | |  |  |  |  | | | Log likelihood ratio (5) = 41.01 | | | |  |  |  |
| * Significant attribute [*P* < 0.05] | |  |  |  |  |  |  | |  | |  |  | |  |  |
| **95% confidence intervals generated using non-parametric bootstrapping (5000 replications) | | | | | | | | |  | |  |  | |  |  |
| ***Marginal rate of substitution values = β-coefficient for attribute/β-coefficient for cost attribute  ^▲^Borderline significant attribute [*P*=0.05]  ^△^Though the cost contribution attribute was not significant for either sample, everything being equal, both samples preferred lower cost contribution. MRS values were calculated using the cost contribution attribute as the denominator to show how participants trade-off cost contribution against the other service attributes. | | | | | | | | |  | |  |  | |  |  |

**Additional file 3: Sub-group analysis: Results from conditional logistic regression model of disabled child (n=9) and parent (n=9) matched-pairs**
